# Supplementary material for: The Notch signaling pathway is a master regulator of CD8+ T cell exhaustion and differentiation during chronic infection
Source: bioRxiv. 2026 Sep 5:2026.09.01.748651. Preprint. [Version 1] doi: 10.64898/2026.09.01.748651 (PMC13684628; doi:10.64898/2026.09.01.748651)
Supplement: Supplementary file 1 [file media-1.pdf]

SUPPLEMENTARY DATA

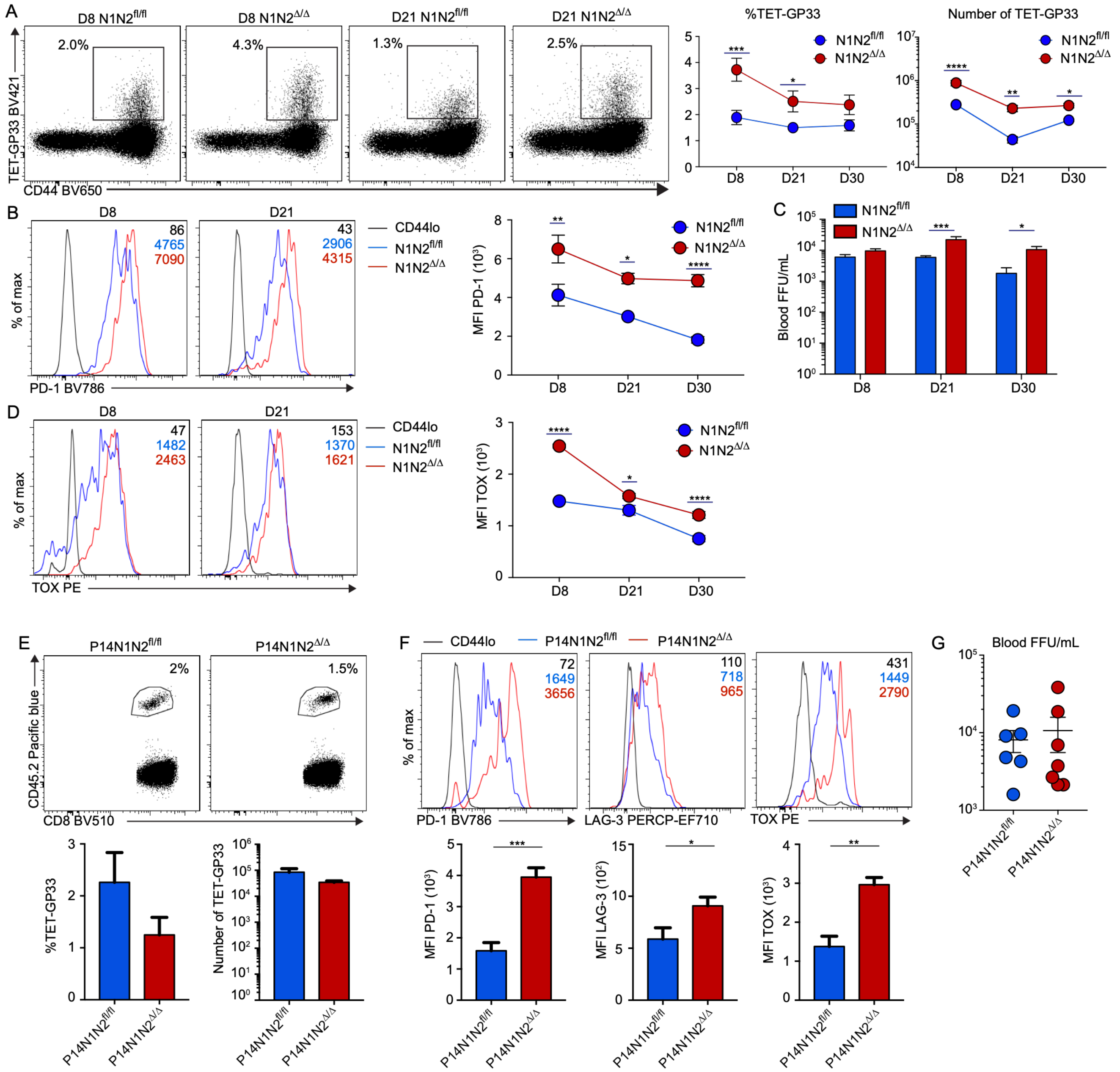

Figure S1. The Notch signaling pathway prevents severe CD8⁺ T cell exhaustion throughout the infection independently of viral load following LCMV clone 13 infection.

(A-C) Mice were infected with LCMV clone 13 and the response in the spleen was characterized at d8, d21 and d30 post-infection by flow cytometry. (**A**) Percentage and numbers of gp33-specific CD8⁺ T cells. Expression of inhibitory receptor PD-1 **(B)** and transcription factor TOX (**C**) on gp33-specific N1N2$^{fl/fl}$ mice and N1N2$^{\Delta/\Delta}$ CD8⁺ T cells over the course of the infection. (**D**) Viral load in the blood was determined using focus-forming assay on MC57G cells. **(E-G)** Adoptive transfer of P14N1N2$^{fl/fl}$ or P14N1N2$^{\Delta/\Delta}$ cells (CD45.2⁺) into B6.SJL recipients 1d before the infection with LCMV

clone 13. The response in the spleen and the viral load were characterized at d30 post-infection by flow cytometry. (**E**) Percentage and numbers of CD45.2$^+$ CD8$^+$ T cells P14N1N2$^{fl/fl}$ and P14N1N2$^{\Delta/\Delta}$ cells. (**F**) Expression of inhibitory receptors (PD-1 and LAG-3) and transcription factor TOX was measured on CD8$^+$CD45.2$^+$ P14N1N2$^{fl/fl}$ and P14N1N2$^{\Delta/\Delta}$ cells. (**G**) Viral load in blood**.** Data are from 2 independent experiment (A-C) and data are representative of 1 from 2 independent experiments (D-F). Unpaired Student's *t* test, with a Welch's correction when applied, was used for two-group comparison. *P<0.05, **P<0.01, ***P<0.001, ****P<0.0001.

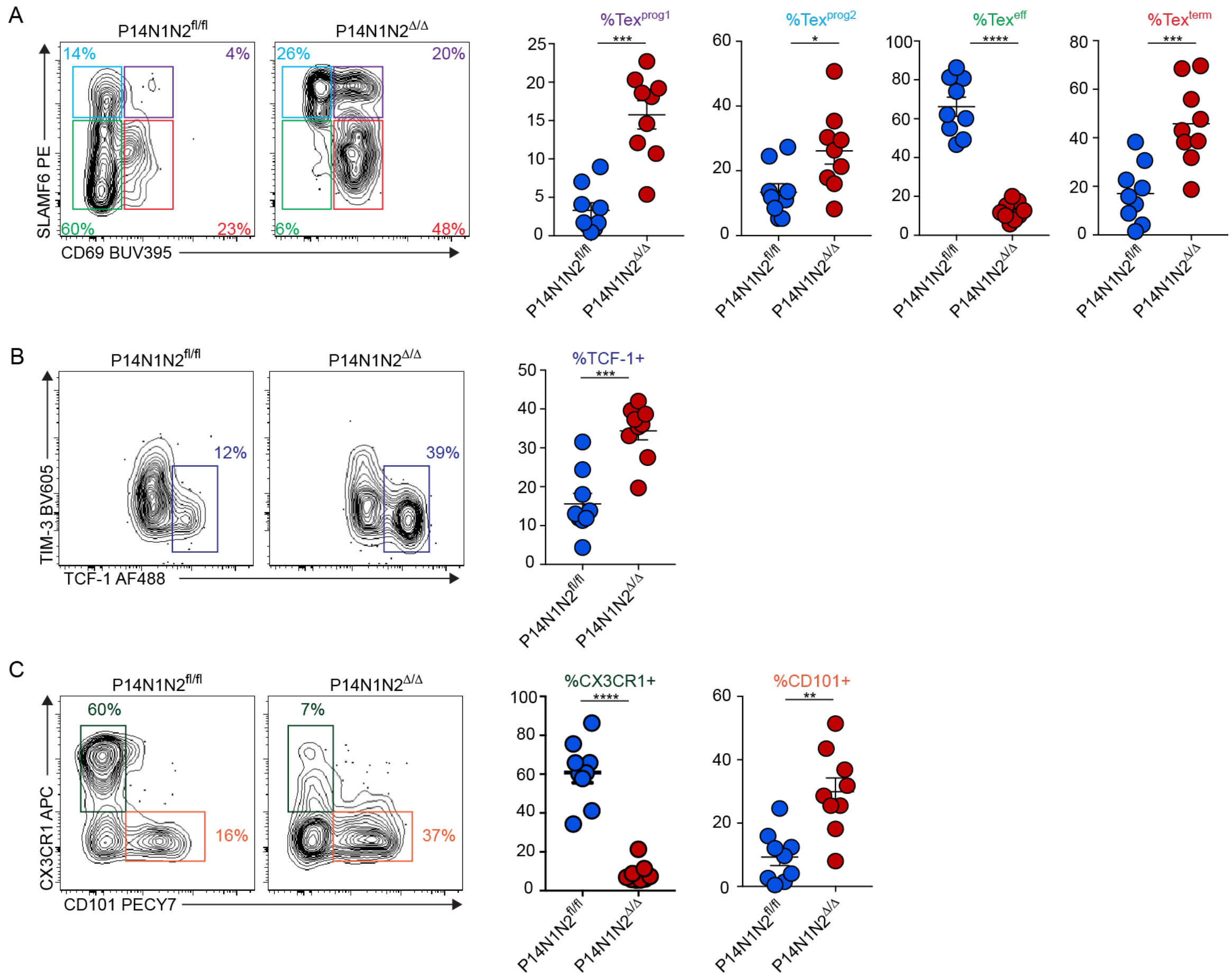

Figure S2. Notch signaling acts as a master regulator of exhausted CD8^{+} T cell differentiation even when the viral loads are normalized.

Adoptive transfer of P14N1N2$^{fl/fl}$ or P14N1N2$^{\Delta/\Delta}$ cells (CD45.2^{+}) into B6.SJL mice was done 1d before LCMV clone 13 infection. The response in the spleen was characterized at d30 post-infection using flow cytometry. Proportion of Tex subsets **(A),** TCF-1^{+} cells (**B**) and CX3CR1^{+} or CD101^{+} cells (**C**) amongst CD45.2^{+} P14N1N2$^{fl/fl}$ or P14N1N2$^{\Delta/\Delta}$ cells. Data are from two independent experiments. Unpaired Student's *t* test, with a Welch's correction when applied, was used for two-group comparison. **P*<0.05, ***P*<0.01, ****P*<0.001, *****P*<0.0001.

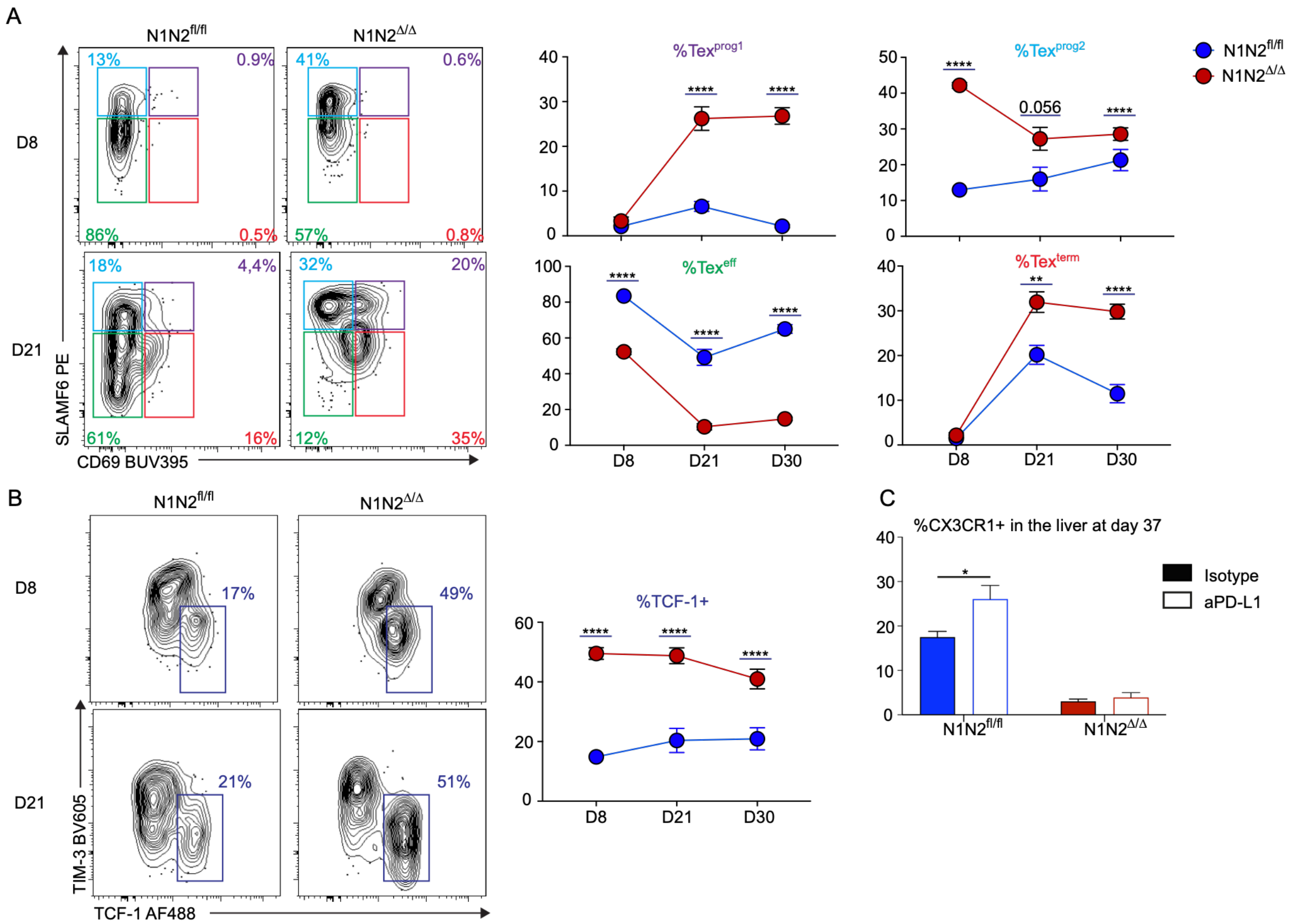

Figure S3. Notch signaling acts as a master regulator of exhausted CD8$^+$ T cell subset differentiation throughout LCMV clone 13 infection.

Mice were infected with LCMV clone 13 and the TET-gp33$^+$ response in the spleen was characterized at different time points by flow cytometry. **(A)** Proportion of Tex subsets within gp33-specific CD8$^+$ T cells from N1N2$^{fl/fl}$ and N1N2$^{\Delta/\Delta}$ mice. (**B**) Proportion and numbers of TCF-1+ cells on gp33-specific CD8$^+$ T cells from N1N2$^{fl/fl}$ and N1N2$^{\Delta/\Delta}$ mice. **(C)** Anti-PD-L1 treatment does not increase the differentiation into Tex eff-like cells in absence of Notch signaling. N1N2$^{fl/fl}$ and N1N2$^{\Delta/\Delta}$ mice were infected with LCMV clone 13 and treated with 0.2 mg of anti-PD-L1 or isotype control every 3 days between day 23 and day 35 post-infection. Proportion of Tex eff-like cells (CX3CR1$^+$) in the liver were determined on gp33-specific CD8$^+$ T cells from N1N2$^{fl/fl}$ and N1N2$^{\Delta/\Delta}$ mice at day 37 post-infection. Data are from two independent experiments. Unpaired Student's *t* test, with a Welch's correction when applied, was used for two-group comparison. $^{**}P<0.01$, $^{****}P<0.0001$.

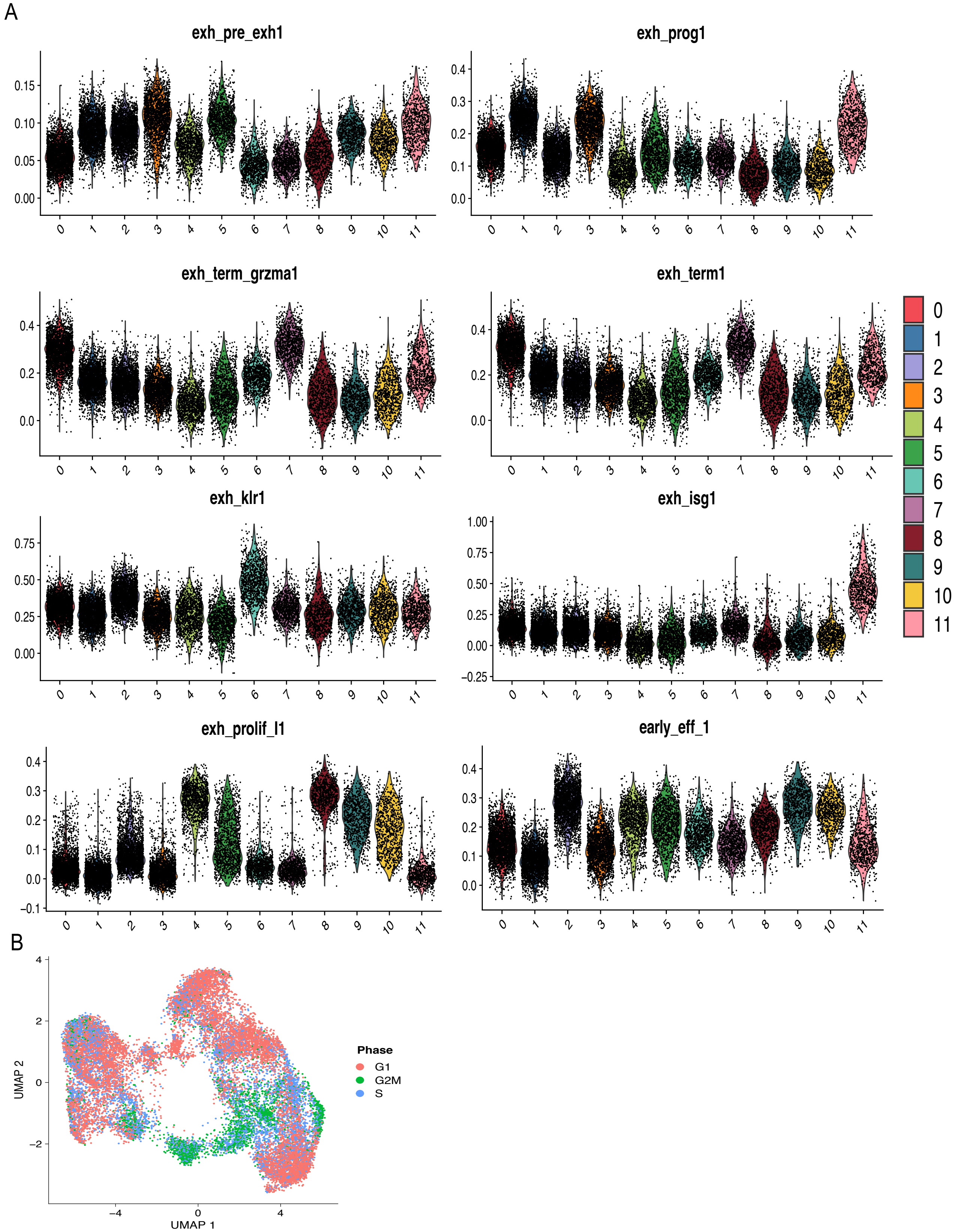

Figure S4. Gene set enrichment analysis and cell cycle status across exhausted CD8⁺ T cell subsets at day 8 and day 21 post–LCMV clone 13 infection.

(A) Violon plot showing the enrichment of gene signature defining CD8 Tex subsets (described by Giles et al., *Nature Immunology* 2022) within the different clusters of our snRNA/ATACseq. Each dot represents an individual nucleus, colored according to cluster identity.
(B) UMAP projection of all nuclei colored by inferred cell cycle phase (G1, S, or G2/M) using mRNA data.

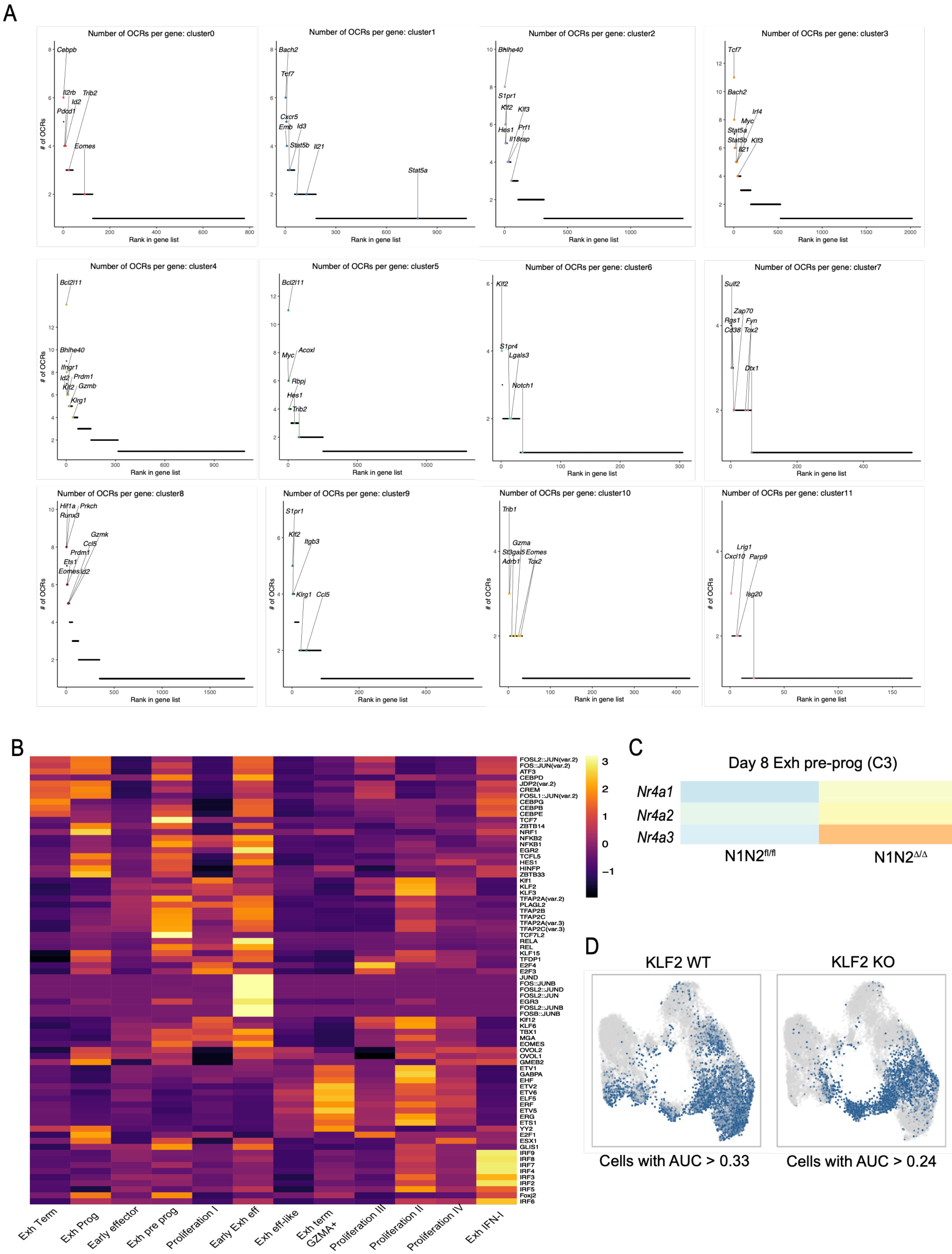
A
Number of OCRs per gene: cluster0
Number of OCRs per gene: cluster1
Number of OCRs per gene: cluster2
Number of OCRs per gene: cluster3
Number of OCRs per gene: cluster4
Number of OCRs per gene: cluster5
Number of OCRs per gene: cluster6
Number of OCRs per gene: cluster7
Number of OCRs per gene: cluster8
Number of OCRs per gene: cluster9
Number of OCRs per gene: cluster10
Number of OCRs per gene: cluster11
# of OCRs
Rank in gene list
Cebpb
Il2rb
Id2
Trib2
Pdcd1
Eomes
Bach2
Tcf7
Cxcr5
Emb
Id3
Stat5b
Il21
Stat5a
Bhlhe40
S1pr1
Klf2
Klf3
Hes1
Prf1
Il18rap
Irf4
Myc
Bcl2l11
Ifngr1
Prdm1
Gzmb
Klrg1
Acoxl
Rbpj
S1pr4
Lgals3
Notch1
Sulf2
Zap70
Rgs1
Fyn
Cd38
Tox2
Dtx1
Hif1a
Prkch
Runx3
Gzmk
Ccl5
Ets1
Itgb3
Trib1
Gzma
St3gal5
Adrb1
Lrig1
Cxcl10
Parp9
Isg20
B
FOSL2::JUN(var.2)
FOS::JUN(var.2)
ATF3
CEBPD
JDP2(var.2)
CREM
FOSL1::JUN(var.2)
CEBPG
CEBPB
CEBPE
TCF7
ZBTB14
NRF1
NFKB2
NFKB1
EGR2
TCFL5
HES1
HINFP
ZBTB33
Klf1
KLF2
KLF3
TFAP2A(var.2)
PLAGL2
TFAP2B
TFAP2C
TFAP2A(var.3)
TFAP2C(var.3)
TCF7L2
RELA
REL
KLF15
TFDP1
E2F4
E2F3
JUND
FOS::JUNB
FOSL2::JUND
FOSL2::JUN
EGR3
FOSL2::JUNB
FOSB::JUNB
Klf12
KLF6
TBX1
MGA
EOMES
OVOL2
OVOL1
GMEB2
ETV1
GABPA
EHF
ETV2
ETV6
ELF5
ERF
ETV5
ERG
ETS1
YY2
E2F1
ESX1
GLIS1
IRF9
IRF8
IRF7
IRF4
IRF3
IRF2
IRF5
Foxj2
IRF6
Exh Term
Exh Prog
Early effector
Exh pre prog
Proliferation I
Early Exh eff
Exh eff-like
Exh term GZMA+
Proliferation III
Proliferation II
Proliferation IV
Exh IFN-I
C
Day 8 Exh pre-prog (C3)
Nr4a1
Nr4a2
Nr4a3
N1N2fl/fl
N1N2Δ/Δ
D
KLF2 WT
KLF2 KO
Cells with AUC > 0.33
Cells with AUC > 0.24

Figure S5. Chromatin accessibility features associated with exhausted CD8⁺ T cell clusters at day 8 and day 21 post–LCMV clone 13 infection.

(A) Ranking of genes based on the number of associated open chromatin regions (OCRs) across individual exhausted $CD8^+$ T cell clusters identified by snRNA/ATAC-seq. For each cluster, genes are ordered according to their rank in the gene list (x-axis), and the corresponding number of OCRs per gene is shown (y-axis), highlighting cluster-specific regulatory programs and chromatin accessibility. **(B)** Heatmap depicting normalized motif accessibility scores across exhausted $CD8^+$ T cell clusters. Color scale indicates relative motif accessibility (z-score), revealing distinct epigenetic landscapes for each cluster. **(C)** Transcription of the TCR-induced genes, *Nr4a1-3*, by Exh pre-porg (cluster 3) cells from $N1N2^{fl/fl}$ and $N1N2^{\Delta/\Delta}$ mice at day 8 post-infection. **(D)** Enrichment of the KLF2 signature (Fargerberg et al. 2025) in Exh pre pro cells (cluster 3) and effector cells (clusters 2, 5 and 6).

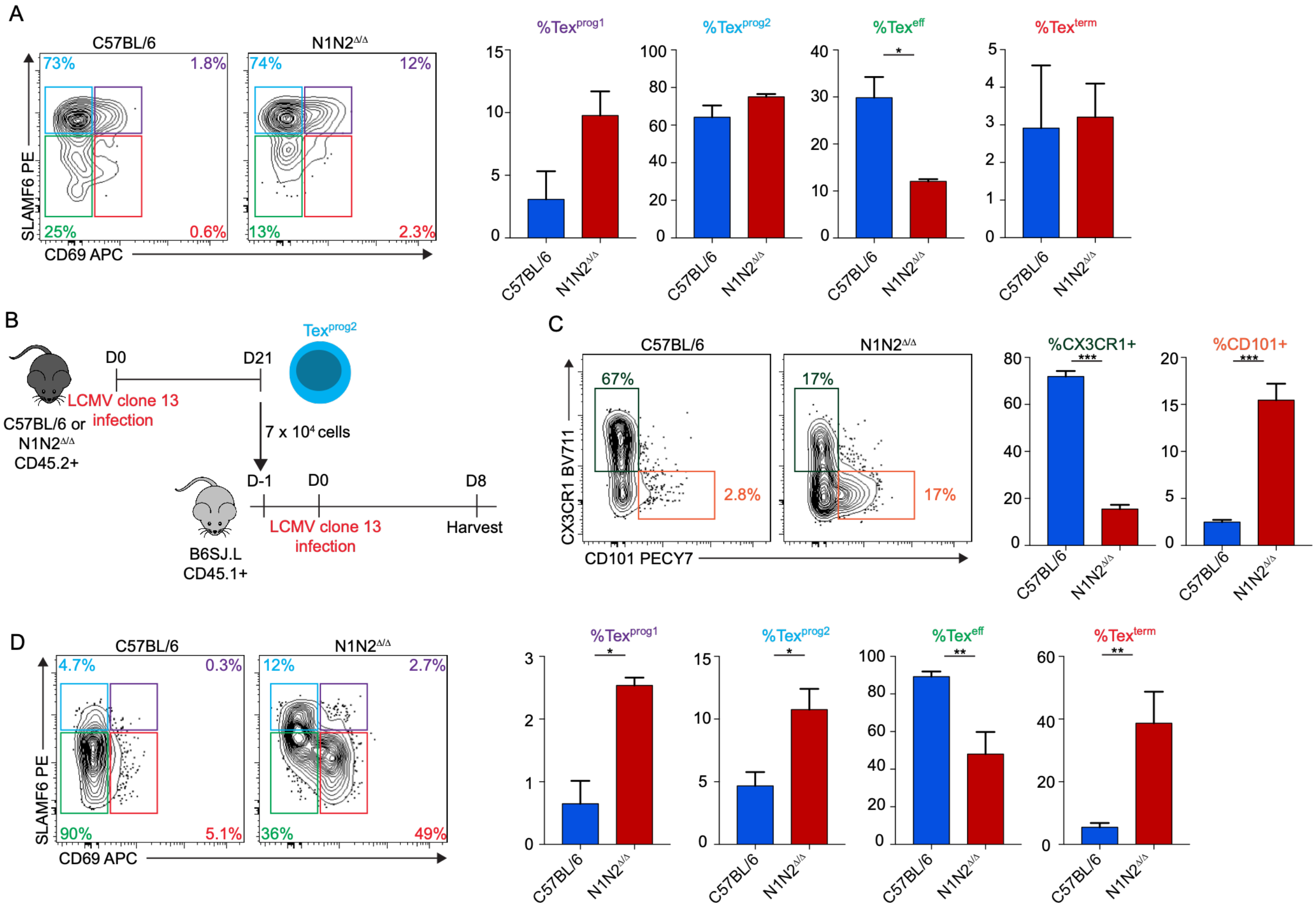

Figure S6. Notch signaling regulates the differentiation of Tex progenitors into Tex effector-like cells after rechallenging with LCMV clone 13.

(A) Adoptively transferred C57BL/6 and N1N2$^{\Delta/\Delta}$ Tex prog2 cells at day 21 post-infection into LCMV clone 13 match-infected B6.SJL recipient mice (CD45.1) (Fig. 6C) where we measured the expression of SLAMF6 and CD69 at day 7 post-transfer. (**B**) Experimental design for the adoptive transfer of day 21 Tex prog2 cells C57BL/6 or N1N2$^{\Delta/\Delta}$ into naive recipients (CD45.1+). Naïve mice were adoptively transferred with sorted cells and infected the next day with LCMV clone 13. Mice were euthanized 8 days post-infection. Proportion of CX3CR1+ cells or CD101+ cells (**C**) and of Tex subsets (**D**) within the adoptively transferred CD8^{+}CD45.2^{+} cells from C57BL/6 and N1N2$^{\Delta/\Delta}$ cells.

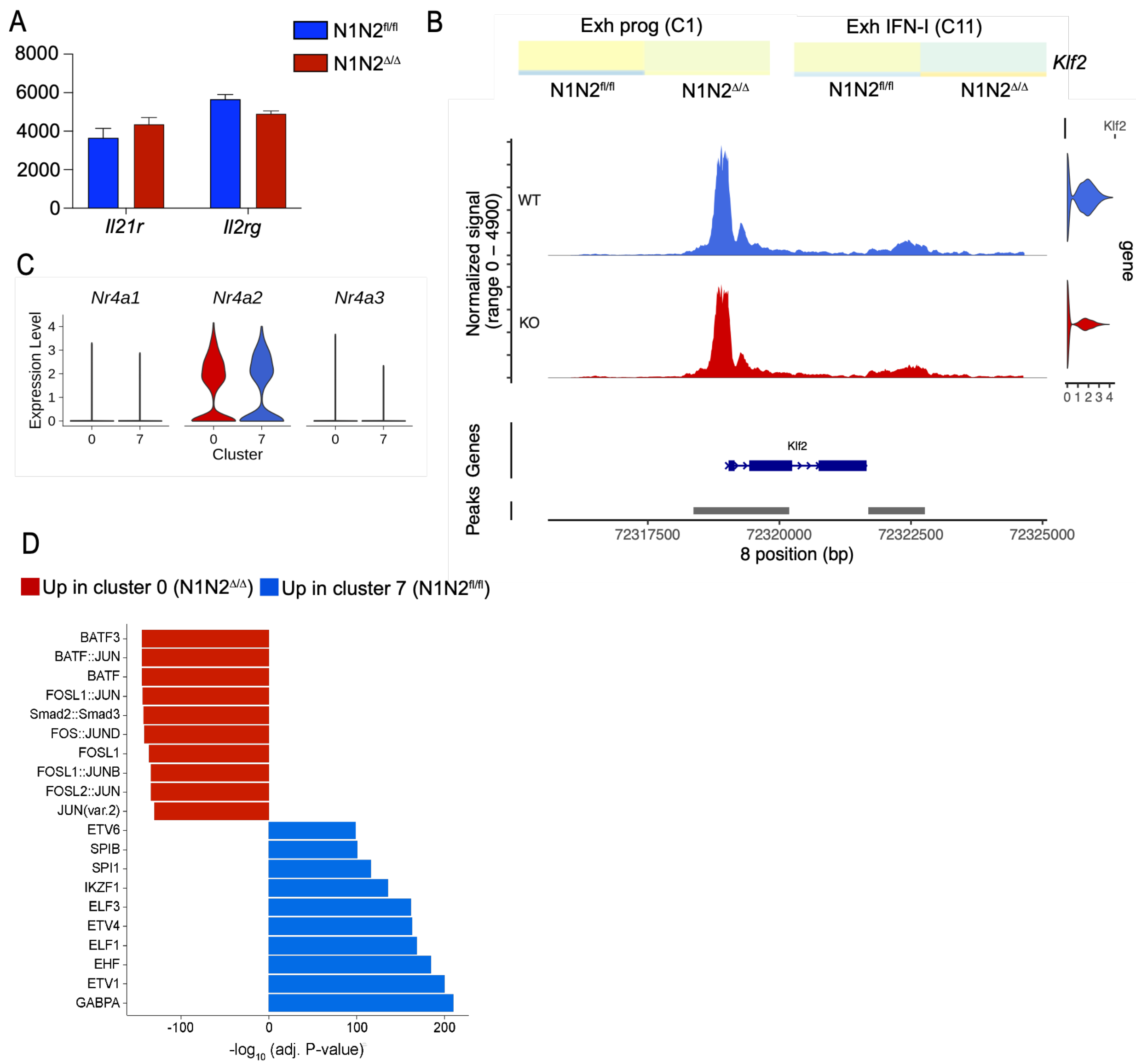

Figure S7. The loss of Notch signaling during the differentiation of exhausted CD8⁺ T cells decreases *Klf2* transcription and gene accessibility while increasing TCR and TGF-β responses.

(A) Notch signaling does not affect transcription of the genes coding for the IL-21 receptor. **(B)** Notch signaling regulates the transcription of *KLf2* and gene accessibility. **(C)** Notch-deficient Tex term cells transcribed more the TCR induced genes *Nr4a1*, *Nr4a2* and *Nr4a3*. **(D)** Smad2::Samd3 motif are enriched within the DARs of N1N2$^{\Delta/\Delta}$ Tex term cells (cluster 0) compared to their WT counterpart.
